# Developmental stage-dependent effects of cardiac fibroblasts on function of stem cell-derived engineered cardiac tissues

**DOI:** 10.1038/srep42290

**Published:** 2017-02-09

**Authors:** Brian Liau, Christopher P. Jackman, Yanzhen Li, Nenad Bursac

**Affiliations:** 1 Department of Biomedical Engineering, Duke University, Durham, NC, USA.

## Abstract

We investigated whether the developmental stage of mouse cardiac fibroblasts (CFs) influences the formation and function of engineered cardiac tissues made of mouse embryonic stem cell-derived cardiomyocytes (mESC-CMs). Engineered cardiac tissue patches were fabricated by encapsulating pure mESC-CMs, mESC-CMs + adult CFs, or mESC-CMs + fetal CFs in fibrin-based hydrogel. Tissue patches containing fetal CFs exhibited higher velocity of action potential propagation and contractile force amplitude compared to patches containing adult CFs, while pure mESC-CM patches did not form functional syncytium. The functional improvements in mESC-CM + fetal CF patches were associated with differences in structural remodeling and increased expression of proteins involved in cardiac function. To determine role of paracrine signaling, we cultured pure mESC-CMs within miniature tissue “micro-patches” supplemented with media conditioned by adult or fetal CFs. Fetal CF-conditioned media distinctly enhanced CM spreading and contractile activity, which was shown by pathway inhibitor experiments and Western blot analysis to be mediated via MEK-ERK signaling. In mESC-CM monolayers, CF-conditioned media did not alter CM spreading or MEK-ERK activation. Collectively, our studies show that 3D co-culture of mESC-CMs with embryonic CFs is superior to co-culture with adult CFs for *in vitro* generation of functional myocardium. Ensuring consistent developmental stages of cardiomyocytes and supporting non-myocytes may be a critical factor for promoting functional maturation of engineered cardiac tissues.

While the developmental origins of cardiac fibroblasts (CFs) continue to be actively studied, a large body of work has described critical roles that these non-myocytes play in heart development, physiology, and disease^1^,^2^. During development, the heart grows via the processes of cardiomyocyte (CM) hyperplasia (proliferation) and hypertrophy (increase in cell size), with the switch from hyperplastic to hypertrophic growth occurring shortly after birth, e.g. neonatal days 4–7 in mice^3^,^4^,^5^,^6^. The proliferation of compact myocardium, a key step in the formation of full-thickness ventricular wall during fetal development, is driven among other cues by CF-secreted paracrine factors^7^,^8^,^9^. In contrast, in adult hearts, CF-secreted factors have been shown to contribute induction of CM hypertrophy in a variety of disease^10^,^11^,^12^,^13^ and non-disease^3^,^14^,^15^ related contexts. The CFs also regulate composition of cardiac extracellular matrix which is one of the main determinants of heart’s structure, function, mechanics, and pathological remodeling^16^,^17^,^18^. Collectively, these findings have indicated that developmental changes in CFs proceed in tandem with changes in CMs, facilitating normal physiological processes and/or mediating pathological remodeling.

In the context of tissue engineering, a variety of non-myocytes (e.g. stromal and vascular cells) from different species and tissues, including cardiac fibroblasts, have been shown to promote the formation and contractile function of engineered cardiac tissues fabricated using decellularized myocardium^19^, polymer scaffolds^20^,^21^, fibrin and collagen-based hydrogels^22^,^23^, and detachable cell sheets^24^. For example, we have recently shown that pure mouse embryonic stem cell-derived cardiomyocytes (mESC-CMs) encapsulated in 3D hydrogel matrix do not form functional tissues *in vitro*, a process that is rescued when mESC-CMs are mixed with small percent of cardiac fibroblasts^22^. Specifically, over the course of 14 days, mESC-CMs in 3D co-culture with neonatal rat CFs became progressively more elongated, interconnected with connexin-43 and N-cadherin junctions, electrophysiologically mature, and capable of uniform and rapid action potential conduction. Thus, while freshly differentiated mESC-CMs are shown to have immature properties similar to mouse outflow tract CMs on embryonic day 12.5 25, 3D culture environment and supporting non-myocytes can promote maturation of mESC-CMs (e.g. cell size, sarcomere structure, active contraction forces, velocity of action potential conduction) to levels characteristic of neonatal mouse myocardium^21^,^22^. Still, it remains unknown if CFs of different developmental stages have distinct effects upon engineered cardiac tissue formation and function, either through direct cell-cell contact or paracrine effects^9^,^26^,^27^,^28^. While engineered tissues containing age-matched CMs and CFs would best mimic developing myocardium, using adult CFs could conceivably accelerate CM maturation by providing an *in vitro* tissue environment more akin to that of adult myocardium.

To explore this question, we employed a same-species setting whereby CFs isolated from E13.5 mouse embryos or adult mouse hearts were co-cultured with purified mESC-CMs to form engineered cardiac tissues (Fig. 1a). Electrophysiological properties of the engineered tissues were assessed by optical recording of intracellular calcium transients, and active (contractile) and passive mechanical properties were studied using isometric force measurement tests. Immunostaining and Western blot analyses were performed to assess structural and protein-expression changes in the engineered tissues caused by the two CF populations. For paracrine studies, we examined the effects of CF-conditioned media on miniature engineered cardiac tissue patches (“micro-patches”) made of mESC-CMs. Selected small molecule inhibitor drugs were applied in this system to further elucidate the intracellular pathways involved in paracrine effects of CFs on mESC-CMs. Collectively, these studies show the distinct effects of cardiac fibroblast age on functional cardiomyogenesis *in vitro* and carry important implications for the field of cell-based cardiac therapy.

## Results

### Phenotype and purity of enzymatically isolated fetal and adult CFs

Although we^27^,^29^ and others^30^ have shown that CFs from neonatal rat cardiac tissue can be purified by differential pre-plating, we found that E13.5 fetal mouse CFs and CMs were similarly adhesive to tissue culture plastic. Thus, as previously described^3^,^31^, we used magnetic-activated cell sorting (MACS) to enrich the percentage of CD90^+^/CD31^−^ fetal CFs from a low purity of 68.1 ± 7.3% to a relatively high purity of 84.5 ± 4.0% (Supplementary Fig. S1). Adult cardiac cells isolated using our enzymatic digestion protocol consisted almost entirely of CD90^+^/CD31^−^fibroblasts (86.9 ± 4.4%), thus obviating the need for further MACS purification. Isolated and purified cells were morphologically homogenous and ubiquitously stained positive for mesodermal marker vimentin, while simultaneously being negative for cardiac (sarcomeric α-actinin), endothelial (VWF), and smooth muscle or myofibroblast (SMA) markers^22^,^26^ (Supplementary Fig. S2).

### CFs modulate the structure and function of engineered cardiac tissue patches in a developmental stage-dependent manner

**Figure 1:**
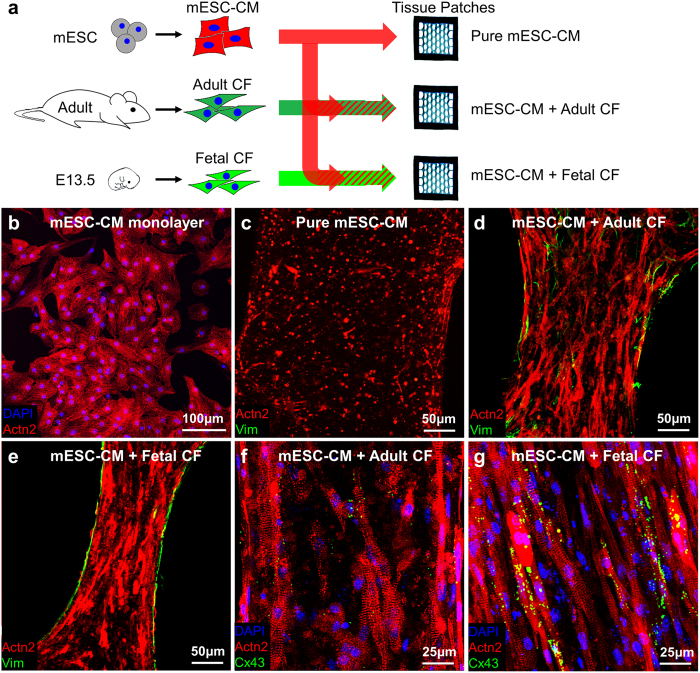
Morphology of co-cultured cardiac tissue patches. (**a**) Schematic of experimental setup showing co-culture of mESC-CMs with CFs from adult or fetal mice in engineered tissue patches. (**b**) Representative mESC-CM monolayer stained for sacromeric α-actinin (Actn2) and DAPI, showing 100% purity of puromycin-selected CMs. (**c**-**e**) Representative tissue patches containing pure mESC-CMs (c), mESC-CMs + adult CFs (**d**), and mESC-CMs + fetal CFs (e), cultured for 14 days, stained for Actn2 and vimentin (Vim). (**f**–**g**) Representative mESC-CM + adult CF (f) and mESC-CM + fetal CF (**g**) tissue patches stained for DAPI, Actn2, and connexin 43 (Cx43).

**Figure 2:**
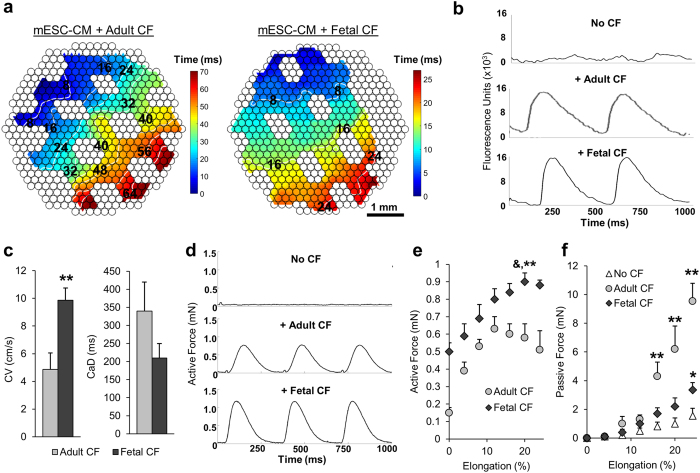
Functional properties of co-cultured tissue patches. (**a**) Representative Ca^2+^ transient activation maps in an mESC-CM + adult CF tissue patch (left) and an mESC-CM + fetal CF tissue patch (right). Isochronous lines demonstrate 8 ms increments in activation time; note differences in temporal color bar scales. (**b**) Ca^2+^ transient traces from the cellular regions of a pure mESC-CM tissue patch (top), an mESC-CM + adult CF co-culture patch (middle), and an mESC-CM + fetal CF co-culture patch (bottom). (**c**) Action potential conduction velocity (CV) and Ca^2+^ transient duration (CaD) in mESC-CM tissue patches cultured with fetal and adult CFs. (**d**) Representative contractile force traces during 3 Hz field stimulation of mESC-CM tissue patches without CFs (top), with adult CFs (middle), or with fetal CFs (bottom). (**e**–**f**) Active (**e**) and passive (**f**) force-length curves in patches made with no CFs, adult CFs, or fetal CFs. **Significantly different between adult CFs and fetal CFs; &, significantly different from adult CF maximum force at different length; *Significantly different from no CF group. N = 6–9 patches per group from 3 independent mESC-CM differentiations, each using independent fetal and adult CF isolations.

Similar to our previous study^22^, the encapsulation of only pure mESC-CMs (Fig. 1b) in tissue patches resulted in minimal gel compaction and rounded cell morphology that persisted throughout the culture duration (Fig. 1c). Addition of CFs yielded hydrogel compaction that was more pronounced for fetal (Fig. 1e) than for adult (Fig. 1d) fibroblasts. Based on histological analysis of nuclei numbers in α-actinin^+^ areas, the percentage of mESC-CMs in 2-week tissue patches decreased slightly from the initial 75% to 59.0 ± 4.8% and 57.8 ± 8.9% for adult and fetal CFs, respectively. Importantly, mESC-CMs in tissue patches co-cultured with fetal CFs appeared to be more spread, aligned, and to have more abundant connexin-43 expression (Fig. 1g) than those co-cultured with adult CFs (Fig. 1f). Similar to our studies using neonatal rat CFs, optical mapping of co-cultured cardiac tissue patches made of mESC-CMs and adult or fetal CFs revealed continuous action potential conduction during point pacing (Fig. 2a). Interestingly, conduction velocity (CV) of patches made with fetal CFs (9.86 ± 0.88 cm/s) was higher than that of patches made with adult CFs (4.87 ± 1.18 cm/s), while Ca^2+^ transient duration was lower (209.2 ± 39.3 ms for fetal vs. 344.9 ± 83.7 ms for adult CFs), but without reaching statistical significance (Fig. 2c, *p* = 0.1). Similar to our previous studies^22^, tissue patches with added CFs (adult or fetal) generated significant contractile forces upon electrical stimulation (Fig. 2d). In contrast, cardiac patches containing only mESC-CMs and no CFs were unable to sustain any action potential propagation in response to point pacing or a near-threshold field shock and did not generate measurable contractile force (Fig. 2b,d). From average force-length relationships (Fig. 2e), tissue patches containing fetal CFs produced significantly higher baseline (0.50 ± 0.06 mN vs. 0.15 ± 0.03 mN) and maximum contractile force (0.90 ± 0.07 mN vs. 0.63 ± 0.09 mN) than those containing adult CFs. Finally, with applied stretch, tissue patches made with adult CFs generated more passive tension than those made with fetal CFs or no-CF controls (9.53 ± 1.26 mN vs. 3.35 ± 0.52 mN vs. 1.54 ± 0.54 mN, at 24% elongation, Fig. 2f).

### Fetal and adult CFs differently affect expression of cardiac function-related proteins

**Figure 3:**
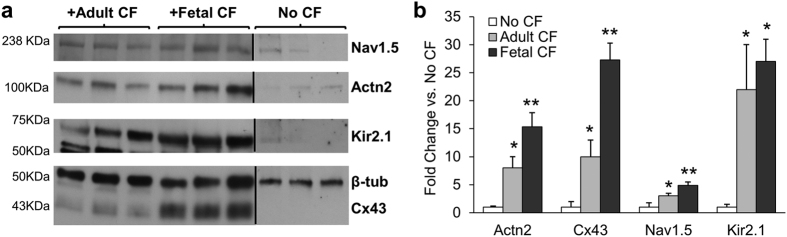
Protein expression in co-cultured tissue patches. (**a**) Representative Western blots for voltage-gated sodium channel (Nav1.5, blot cropped to show region near 238 kDa), sarcomeric α-actinin (Actn2, cropped to 100 kDa region), inward rectifying potassium channel (Kir2.1, cropped to 50–75 kDa), connexin-43 (Cx43, cropped to 43–50 kDa region) and β-tubulin (β-tub, loading control) from tissue patches with pure mESC-CMs (No CF) or co-culture of mESC-CMs with adult or fetal CFs. Each band represents protein isolate from an independent tissue patch. (**b**) Relative expression of indicated proteins per β-tubulin, normalized to no CF group, quantified from non-saturated Western blots with samples from identical experiments processed in parallel. *Significantly different from patches without CFs; **Significantly different from patches containing adult CFs. N = 4–6 patches per group.

By performing Western blot analysis, we found that the mESC-CM patches containing either fetal or adult CFs exhibited significantly increased expression of cardiomyocyte proteins relevant for electrical and mechanical function, compared to patches made of pure mESC-CMs (Fig. 3a). Furthermore, fetal CFs increased cardiac protein expression more than adult CFs. Specifically, tissue patches containing fetal CFs had 1.92-fold higher expression of sarcomeric α-actinin (Actn2), a microfilament protein vital for the attachment of actin filaments to the Z-lines in myocytes, as well as 2.73-fold and 1.63-fold higher expression of connexin-43 and Nav1.5, respectively, compared to patches containing adult CFs. No significant difference was found between adult and fetal CF co-culture for the expression of Kir2.1 (Fig. 3b). Collectively, these results demonstrated that 3D co-culture of CFs with mESC-CMs significantly enhanced the expression of cardiac structural and functional proteins, and that this effect was more pronounced when using fetal compared to adult CFs.

### Conditioned media from fetal and adult CFs differently increase cardiomyocyte spreading in engineered micro-patches

**Figure 4:**
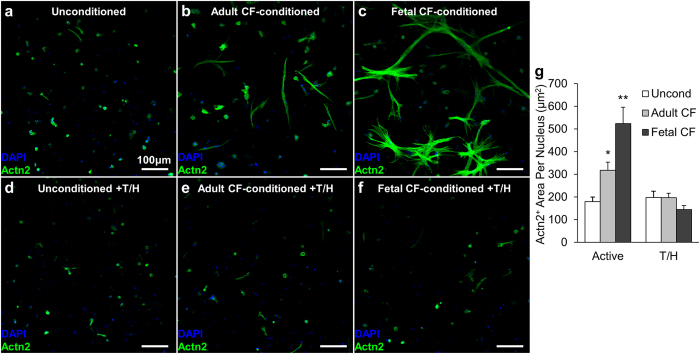
Cell spreading in mESC-CM micro-patches conditioned by CF paracrine factors. (**a**–**c**) Representative immunostainings of cardiac micro-patches cultured for 2 weeks in unconditioned serum-free media (**a**) or media conditioned for 48 hours by adult CFs (**b**), or fetal CFs (**c**), stained for DAPI and sarcomeric α-actinin (Actn2). **(d**–**f)** mESC-CM micro-patches cultured in indicated media subjected to protein deactivation by trypsin and heat (T/H). (**g**) Actn2^+^ area per nucleus from immunostaining images was quantified as the index of cell size. *Significantly different from unconditioned; **significantly different from adult CF-conditioned; N = 6 micro-patches per group.

To assess the effects of paracrine signaling on structural and functional properties of mESC-CMs cultured in a 3D patch environment, we fabricated thin 3D micro-patches that contained small numbers of sparsely encapsulated pure mESC-CMs expected to be uniformly accessible by soluble factors from serum-free conditioned media (Supplementary Fig. S3). Consistent with the initial results (Fig. 1c), mESC-CMs in the micro-patches remained viable but rounded (Fig. 4a). Addition of CF-conditioned media yielded spreading of mESC-CMs with stronger effects induced by fetal than adult CF paracrine factors (Fig. 4b–c). Notably, protein deactivation of CF-conditioned media by trypsin and heating abolished these effects (Fig. 4d–g), demonstrating that the paracrine factors responsible for CM spreading were secreted proteins.

### Conditioned media from fetal and adult CFs differently enhance the contractile activity of mESC-CMs

**Figure 5:**
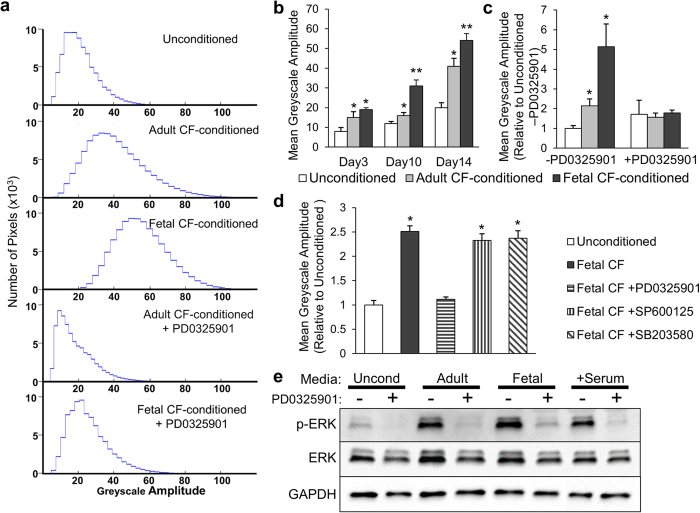
Role of CF paracrine factors and MEK/ERK signaling in amplitude of spontaneous contractions in mESC-CM micro-patches. (**a**) Histograms showing the range of pixel greyscale amplitudes in videos of spontaneous contractions within cardiac micro-patches, cultured for 14 days in the absence (top) or presence of adult (2^nd^ row) or fetal (3^rd^ row) CF-conditioned media, or CF-conditioned media with MEK1/2 inhibitor PD0325901 (adult in 4^th^ row, fetal in 5^th^ row). (**b**) Change in contraction amplitude of CF-conditioned mESC-CM micro-patches with time of conditioning. (**c**) Contraction amplitude of mESC-CM micro-patches cultured for 14 days in CF-conditioned media, with or without PD0325901. (**d**) Contraction amplitude of mESC-CM micro-patches cultured for 14 days in fetal CF-conditioned media supplemented with inhibitors of MEK 1/2 (PD0325901), JNK (SP600125), or p38 (SB203580). *Significantly different from unconditioned patches; **Significantly different from adult CF-conditioned patches. N = 6 micro-patches per group. **(e)** Western blot analysis of phospho-ERK1/2 (p-ERK), total ERK1/2 (ERK), and GAPDH as loading control in mESC-CM patches cultured for 7 days in indicated media with or without MEK inhibitor PD0325901. Stimulation with 15% FBS (+Serum) is included as positive control for phosphorylated ERK1/2. Blots cropped to show region corresponding to predicted molecular weights of ERK1/2 (42–44 kDa) and GAPDH (36 kDa).

Visual inspection of micro-patches suggested that the amplitude of their spontaneous contractions was greater in the presence of conditioned media from fetal than adult CFs (Supplementary Video 1). To quantify the contraction amplitudes in different micro-patches, we recorded videos of spontaneous contractions and measured grayscale value deviation in individual pixels over a 5 sec recording period. The patches containing mESC-CMs that contracted more vigorously showed higher values of greyscale fluctuation (Fig. 5a). We averaged the amplitudes of greyscale signals from all pixels to obtain a parameter that quantified the intensity of contraction observed in each micro-patch. From this analysis, we found that the contraction intensity in all micro-patches increased over time in culture, with fetal CF-conditioned media inducing larger increase than adult CF-conditioned media (Fig. 5b).

### Effects of CF-conditioned media on mESC-CM micro-patches depends on MEK-ERK signaling

After observing that media conditioned by fetal CFs exhibited strongest effect on spreading and contractile activity of mESC-CMs, we sought to reveal potential signaling pathways underlying this finding. Micro-patches cultured with CF paracrine factors were simultaneously incubated with small molecule inhibitor drugs targeted against specific intracellular pathways, and assessed for contractile activity to provide a live readout. The addition of MEK1/2 inhibitor PD0325901 significantly decreased the intensity of spontaneous contractions in patches conditioned with adult or fetal CFs to the same level as in unconditioned patches (Fig. 5a,c), an effect that was not observed during application of other small molecules (Supplementary Table S5), including JNK and p38-MAPK inhibitors (Fig. 5d). Histological analysis indicated that MEK inhibition with PD0325901 blocked the enhanced CM spreading from fetal CF-conditioned media (Fig. S4), which was also observed for CMs in micro-patches cultured in serum-containing media (Fig. S5). Finally, assessment of ERK1/2 phosphorylation by Western blotting revealed that while MEK-ERK activity was low in the presence of unconditioned media, it was significantly increased by CF paracrine factors and abolished by PD0325901 (Fig. 5e). Taken together, these results strongly supported a critical role of MEK-ERK signaling in the enhanced spreading and contractile activity of CMs in response to CF paracrine factors.

### Effects of CF-conditioned media on mESC-CMs in 2D monolayer cultures

**Figure 6:**
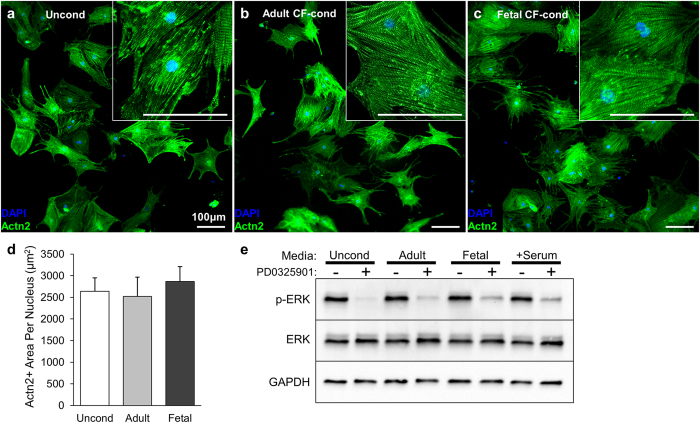
Effect of CF paracrine factors on spreading and MEK/ERK signaling of mESC-CMs cultured in 2D monolayers. (**a**–**c**) Representative immunostainings of mESC-CM monolayers in unconditioned serum-free media (**a**), or media conditioned for 4 days by adult CFs (**b**) or fetal CFs (**c**), stained for nuclei (DAPI) and sarcomeric α-actinin (Actn2). Insets contain higher magnification images demonstrating organized sarcomere structure. (**d**) Actn2^+^ area per nucleus for mESC-CMs shown as the index of cell size. (n = 4 monolayers per group). (**e**) Western blot analysis of phospho-ERK1/2 (p-ERK), total ERK1/2 (ERK), and GAPDH as loading control in mESC-CMs monolayers cultured for 4 days in indicated media with or without MEK inhibitor PD0325901. Stimulation with 15% FBS (+Serum) is included as positive control for phosphorylated ERK1/2. Blots cropped to show region corresponding to predicted molecular weights of ERK1/2 (42–44 kDa) and GAPDH (36 kDa).

We also assessed the effects of adult and fetal CF paracrine factors on mESC-CMs cultured in 2D monolayers. In contrast to the rounded morphology in 3D culture, pure mESC-CMs cultured on fibronectin-coated 2D surface spread and formed well-organized sarcomeric structures in unconditioned serum-free media (Fig. 6a). Application of adult or fetal CF-conditioned media did not affect CM sarcomeric organization (Fig. 6b,c) or size (Fig. 6d) relative to unconditioned control. Moreover, CF paracrine factors or serum conditioning in 2D cultures did not enhance ERK phosphorylation, which, in contrast to 3D culture (Fig. 5e), was already elevated in control conditions (Fig. 6e).

## Discussion

Although there has been a wealth of literature describing the interaction between CFs and CMs^10^,^32^,^33^,^34^,^35^,^36^, in most cases the focus has been on the compensatory or pathological effects of CFs in disease states including myocardial infarction and heart failure. In the context of heart development, different studies have described signaling events between non-cardiac cells and CMs that contribute to cardiac organogenesis. For example, the primitive streak and visceral endoderm provide commitment signals to cardiac progenitor cells^37^, which can enhance the capacity of mouse and human ESCs to differentiate towards CMs^38^. Similarly, the endocardium and epicardium are known to signal to the myocardium via several paracrine pathways involving neuregulin^39^,^40^, FGF^41^, endothelin^42^, and retinoic acid^43^, supporting either CM specification or maturation. To the best of our knowledge, there has been only one previous study examining the developmental stage-dependent effects of CFs on CMs^3^. In 2D co-culture studies, Ieda *et al*. showed that fetal (but not adult) murine CFs induced a potent proliferative effect on embryonic CMs. This effect appeared to be mediated by CF-secreted HB-EGF and dependent on β1-integrin engagement of CMs. On the other hand, the presence of adult (and to a lesser extent, fetal) CFs tended to increase the apparent size of CMs. More recently, adult CFs were found to deteriorate the electrophysiological function of mESC-CMs in 2D co-culture^44^.

In our study, we chose to focus on the functional consequences of fibroblast-myocyte signaling in 3D engineered tissues, that compared to traditional 2D cultures are more representative of the native cardiac tissue environment^45^. We discovered that CF developmental stage has a strong effect on the structure, function, and protein expression of mESC-CMs in engineered cardiac tissues. Specifically, while CM fractions in tissue patches containing adult and fetal CFs were comparable, co-culture with fetal vs. adult CFs resulted in superior cardiac function as evidenced by 2.02-fold higher conduction velocity and 1.43-fold higher maximum force of contraction. This enhancement of functional properties was associated with a 2.73-fold, 1.63-fold, and 1.92-fold higher connexin-43, Nav1.5, and sarcomeric α-actinin protein expression, respectively. Overall, these findings suggested that fetal CFs induced a greater degree of functional maturation in engineered cardiac tissues than adult CFs. Our studies also showed that patches containing adult CFs generated significantly higher passive tension, consistent with findings that fibroblasts become stiffer with age^46^.

CFs are known to signal to CMs extensively through a variety of paracrine mechanisms both in development^3^ and disease^10^,^13^,^35^. Therefore, we further studied if the functional differences found in direct 3D co-cultures of mESC-CMs with fetal vs. adult CFs could be attributed to age-dependent differences in fibroblast paracrine action. We observed that paracrine factors secreted by fetal CFs enhanced mESC-CM spreading and intensity of spontaneous contractions more than the adult CF factors. While the observed effect of fibroblast paracrine factors on CMs is in agreement with reports mESC-CMs in avitalized myocardial tissue^19^, the dependence of these paracrine actions on the age of fibroblasts has not been previously studied. Assuming that the stiffness of extracellular matrix in which sparse mESC-CMs were embedded in our study did not change by adding paracrine factors, the more vigorous contractions in the presence of CF-conditioned media were likely a result of increased cardiomyocyte force production or improved electrical coupling due to more abundant cell-cell contacts. Thus, paracrine signaling, at least in part, contributed to the age-dependent effects of CFs on the structural and functional assembly of hydrogel-encapsulated mESC-CMs into a dense, contractile 3D syncytium *in vitro*.

To gain better insight in mechanisms of the observed paracrine effects, our strategy was to apply specific inhibitors of individual pathways known to regulate CM development and hypertrophy, and establish which of these inhibitors reduce the observed age-dependent effects of CF-conditioned media on CMs. Using this strategy, we found that differential paracrine signaling of fetal vs. adult CFs to cardiac myocytes predominantly involved the MEK-ERK pathway. Although ERK has been shown to contribute to pathological hypertrophy^14^, it has been also established as a highly important mediator in normal cardiac growth and development^47^,^48^. Indeed, cardiac-specific overexpression of MEK led to an increase in mouse heart-to-body weight ratio and contractile performance, but did not result in any pathology or premature death^49^. On the other hand, p38 and JNK are specialized mediators of stress or injury^50^, and are commonly known as stress-activated protein kinases resulting in pathological outcomes. Notably, our small molecule experiments showed that unlike MEK1/2 inhibition, the inhibition of p38 or JNK could not reverse the effects of fetal CF-conditioned media on mESC-CMs. These results, in conjunction with the observed benefits on tissue patch electrical and contractile function, suggest that fetal CF paracrine factors exerted structural and functional changes in mESC-CMs characteristic of physiological maturation.

Interestingly, CF-conditioned media failed to increase mESC-CM size and MEK-ERK signaling in standard monolayer cultures, which (in contrast to 3D culture) supported spreading and sarcomere formation of pure CMs in unconditioned, serum-free media. High background levels of MEK-ERK signaling, potentially induced by strong activation of integrins^51^,^52^,^53^,^54^ on non-physiologically stiff 2D substrate^55^,^56^,^57^, have likely masked the effects of CF paracrine factors observed in 3D. Further investigations are warranted to determine whether CF paracrine factors activate the MEK-ERK pathway in 3D cultured mESC-CMs directly or indirectly, by altering cell-matrix interactions.

In summary, we showed that fetal (E13.5) murine cardiac fibroblasts are better able to support the *in vitro* engineering of mESC-CM derived myocardium compared to adult cardiac fibroblasts. Through secreted proteins and effects on tissue remodeling, fetal CFs promoted both formation and function of engineered mESC-CM tissues. Importantly, these results suggest that the recreation of cellular composition consistent with the developmental stage of cardiomyocytes is beneficial for the engineering of 3D functional myocardium, a concept that remains to be explored for human ESC and iPSC-derived cardiomyocytes. Furthermore, our studies warrant additional investigations of how developmental stage-dependent changes in non-myocyte phenotype combined with changes in mechanical load^24^,^58^,^59^, tissue stiffness^60^,^61^,^62^, and soluble molecules^63^,^64^,^65^,^66^,^67^ govern generation and functional maturation of engineered cardiac tissues.

## Methods

Detailed description of methods is provided in the Supplementary Material.

### Isolation of CFs from fetal and adult mice

Methods involving vertebrate subjects were performed in accordance with the Guide for the Care and Use of Laboratory Animals (8^th^ edition) published by the U.S. Institute for Laboratory Animal Research. Experimental protocols used in this study were approved by the Duke University Institutional Animal Care and Use Committee (IACUC) under protocol #A164-15-05. Cells were isolated from the hearts of day 13.5 mouse embryos (E13.5) and from the ventricles of the pregnant mothers by serial enzymatic digestions. The fetal murine cardiac cells were sorted for CD31−/CD90 + fibroblasts by two rounds of magnetic activated cell sorting (MACS), while dissociated adult cardiac cells did not undergo MACS due to sufficient fibroblast purity (Supplementary Fig. S1). The fractions of fibroblasts and endothelial cells within the cardiac cell population were determined by fluorescent activated cell sorting (FACS) analysis for CD31 + /CD90 + endothelial cells and CD31−/CD90 + fibroblasts. The primary CFs were seeded on gelatin-coated tissue culture flasks and passaged once prior to co-culture or conditioned media experiments.

### Fabrication and functional testing of cardiac tissue patches

Pluripotent stem cell-derived cardiac tissue patches were fabricated and analyzed for functional properties as previously described^22^,^68^,^69^. Briefly, mESC-derived cardiomyocytes (mESC-CMs) were differentiated using an embryoid body method and purified based on expression of puromycin resistance gene under α-myosin heavy chain promoter. Engineered tissue patches were created by encapsulation of mESC-CMs in 120 μl of fibrin-based hydrogel (Supplementary Table S3, final density 6.5 × 10^6^ cells/ml) and casting into microfabricated PDMS tissue molds. For co-cultured tissue patches, fetal or adult murine CFs were mixed with the mESC-CMs before encapsulation in a 1:3 ratio. After two weeks of patch culture in serum-free media (Supplementary Table S4), intracellular Ca^2+^ transients were optically mapped using Rhod2-AM dye to determine Ca^2+^ transient duration and conduction velocity^22^,^68^. Passive tension and active (contractile) force in response to electric field stimulation were measured at different tissue elongations^22^,^69^,^70^,^71^.

### Western Blot

After assessment of electrophysiological and mechanical function, cell lysates collected from co-cultured tissue patches were analyzed by Western blot for cardiac functional markers Nav1.5 (fast Na^+^ channel), Kir2.1 (inward rectifier K^+^ channel), Cx43 (Connexin-43), and sarcomeric α-actinin (SAA). Densitometry was performed on the obtained bands using ImageJ.

### Conditioned media studies in cardiac tissue “micro-patches”

To efficiently study soluble factors involved in functional maturation of CMs within 3D engineered tissues, we generated miniature cardiac tissue “micro-patches” made of pure mESC-CMs. Specifically, circular nylon rings were filled with 35 μL of hydrogel solution (Supplementary Table S3) containing 7 × 10^4^ CMs and cultured in serum-free media (Supplementary Table S4) for up to two weeks. For conditioned media studies, media in micro-patches were replaced every other day with serum-free media conditioned by adult or fetal CFs for 48 h. In addition, some micro-patches were cultured for two weeks in the presence of small molecule inhibitors (Supplementary Table S5), including PD0325901, SP600125, and SB205380 to inhibit MEK 1/2, JNK, or p38 MAPK pathway, respectively. To assess the amplitude of spontaneous contractions of cardiomyocytes within micro-patches, light microscopy videos were recorded in a heated/CO2 perfused chamber and analyzed for fluctuations in the greyscale intensity of individual pixels. To assess MEK-ERK activation in pure mESC-CM patches, cell lysates were collected after one week culture in conditioned media and analyzed by Western blotting for phosphorylated and total ERK1/2.

### Quantification of CM spreading

Micro-patches were fixed with paraformaldehyde and stained with sarcomeric α-actinin antibody and DAPI for nuclear stain. Confocal microscopy images were analyzed with custom MATLAB software to determine the amount of cellular α-actinin^+^ area per nucleus, which was taken as a quantitative measure of CM spreading within micro-patch.

### Monolayer culture of mESC-CMs

Differentiated mESC-CMs were seeded onto fibronectin-coated Aclar^®^ coverlsips at a low density of 25,000 cells per cm^2^ for immunofluorescent assessment of cell spreading, or at a density of 100,000 cells per cm^2^ for Western blot analysis of ERK phosphorylation. Monolayers were cultured in the indicated media for a period of 4 days prior to fixation or protein collection.

### Statistical Analysis

Data are expressed as mean ± standard error of the mean (SEM), and differences among groups were compared by one-way analysis of variance (ANOVA) with Tukey’s *post hoc* test, with statistical significance defined as p < 0.05.

## Additional Information

**How to cite this article**: Liau, B. *et al*. Developmental stage-dependent effects of cardiac fibroblasts on function of stem cell-derived engineered cardiac tissues. *Sci. Rep.* **7**, 42290; doi: 10.1038/srep42290 (2017).

**Publisher's note:** Springer Nature remains neutral with regard to jurisdictional claims in published maps and institutional affiliations.

## Supplementary Material

Supplementary Information

Supplementary Video 1
