# Supplementary material for: Developmental stage-dependent effects of cardiac fibroblasts on function of stem cell-derived engineered cardiac tissues
Source: Sci Rep. 2017 Feb 9;7:42290. doi: 10.1038/srep42290 (PMC5299411; doi:10.1038/srep42290)
Supplement: Supplementary Information [file srep42290-s1.pdf]

SUPPLEMENTARY INFORMATION

Developmental stage-dependent effects of cardiac fibroblasts on function of stem cell-derived engineered cardiac tissues

Brian Liau[1,#], PhD, Christopher P Jackman[1,#], BS, Yanzhen Li[1], MS, and Nenad Bursac[1,]*, PhD

[1]Department of Biomedical Engineering, Duke University, Durham, NC

[#]Equally contributing authors

* Corresponding Author:

Prof. Nenad Bursac

101 Science Dr.

Room 1427, Fitzpatrick CIEMAS

Durham, NC 27708

USA

Fax: 919-684-4488

Phone: 919-660-5510

Email: nbursac@duke.edu

Detailed Methods

mESC-CM differentiation and purification

mESC-CMs were derived using previously described methods[1]. mESCs expressing puromycin N-acetyl-transferase under a mouse Myh6 promoter were aggregated into embryoid bodies by culturing 4 x 10^6 cells in suspension in a non-adherent 15 cm culture dish coated with 50 mg/ml poly-hydroxyethylmethacrylate (pHEMA). On day 8 of differentiation, mESC-CMs were purified by 5 additional days of culture with 5 µg/ml puromycin. On day 13 of differentiation, embryoid bodies were dissociated by serial digestion with 0.05% trypsin (4 x 20 minutes, 37 °C, 75 RPM orbital shaking), and freshly dissociated mESC-CMs were cultured in tissue patches.

Isolation of adult murine cardiac fibroblasts

Adult CD-1 mice were anesthetized by isofluorane and euthanized by resection of both cardiac ventricles. The ventricular tissue was immediately washed in PBS to remove residual blood, and then finely minced to slurry (< $1mm^3$). The slurry was collected in a 50ml conical tube, and subjected to 4 x 20 min serial digestions at 37°C with a solution containing 0.5mg/ml collagenase type II and 0.5mg/ml trypsin in 1x ADS buffer[2] (Table S1) which had been adjusted to pH of 7.4 using 1N NaOH or HCl, and to osmolarity of 285-300 mOsm using sucrose. At the end of each digestion, liberated cells were aspirated as a supernatant together with the enzyme solution and placed on ice in a fresh 15ml conical tube with an equal volume of serum-containing medium (Table S2). All cells were collected and pelleted by centrifugation at 500 *g* for 5 min, resuspended in fresh medium and allowed to attach to a gelatin-coated tissue culture flask overnight. Cells were expanded on gelatin without passaging for four days in serum-containing medium (Table S2) and then trypsinized and frozen for storage.

Isolation of fetal murine cardiac fibroblasts

Fetal cardiac fibroblasts were isolated from E13.5 day CD-1 mouse embryos. Following euthanasia, uteri from the mother mice were removed to allow isolation of embryos, numbering approximately 10 per mouse. By using a dissection microscope and a pair of fine-point Dumont forceps (Fine Science Tools), hearts were removed from each embryo and then rapidly digested by four 3-min serial digestions using a solution of 2mg/ml collagenase type II (Worthington Biochemicals) in ADS buffer with mechanical disruption. Liberated cells were pelleted at 500 *g*, resuspended in fresh medium, and allowed to attach to gelatin-coated tissue culture flasks. Cells were grown without passaging for four days in serum-containing medium (Table S2) and then trypsinized and frozen for storage.

Magnetic activated cell sorting (MACS)

Freshly isolated fetal murine cardiac cells (consisting of a mix of cardiac myocytes and non-myocytes) were cultured in gelatin-coated tissue culture flasks for 4 days. Cells were then detached by treatment with 0.05% Trypsin-EDTA (Invitrogen), pelleted at 500 *g*, and resuspended in 170µl of a solution consisting of 20% FBS in DPBS + 10U/ml DNase I (Worthington Biochemicals; "MACS buffer"). The cells were incubated with 30µl CD31 beads (Miltenyi Biotec) for 30 min at 4°C, washed twice and passed through a magnetic column (Miltenyi Biotec) to deplete endothelial cells. The flow-through was then pelleted, resuspended in MACS buffer and incubated with CD90.2 beads (Miltenyi Biotec), thus enabling cardiac fibroblasts to be retained once the cells were passed through a second magnetic column. Cardiac fibroblasts were then recovered by washing the column with MACS buffer, and were used directly in co-culture and paracrine experiments.

Fluorescent activated cell sorting (FACS) analysis

Cell fractions were resuspended in MACS buffer and incubated with CD90.2-PE and CD31-APC antibodies (BD Biosciences) for 30 min at 4°C. They were then washed twice in MACS buffer and sorted

using a BD CANTO-II Analyzer. Viable cells were gated based on forward and side scattering, and determined to be positive or negative for the respective cell surface markers by using unstained cells as a negative control. Positive controls used were EL-4 neuroblastoma and MS-1 endothelial cell lines for CD90.2 and CD31, respectively.

Optical mapping of Ca2+ transients

Optical mapping was performed as previously described[1,3]. Tissue patches were incubated in 5 μM Rhod-2 AM dissolved in Media 199 with 0.02 % Pluronic F-127 (45 minutes, 37 °C), washed in Media 199, then incubated for 15 minutes in Media 199 to allow intracellular de-esterification of the Rhod-2 AM. Tissue patches were then maintained in 37 °C Tyrode's solution with 10 μM blebbistatin during optical mapping. Tissue patches were stimulated at a rate of 2 Hz by a bipolar platinum electrode positioned at the periphery of the patch, and fluorescence was recorded through a 4X microscope objective by a 504-channel photodiode array. Conduction velocities were calculated using the difference in activation times between adjacent channels, and Ca^{2+} transient duration (CaD) was measured at 80% recovery. Acellular pores in the tissue patch (regions of low signal intensity) were not included in the analysis.

Isometric force testing

To measure isometric contractile force[1,4], the frame of the tissue patch was pinned to a PDMS block attached to a force transducer. Field stimulus was applied at a rate of 3 Hz by parallel platinum electrodes. The tissue was elongated in 4% increments above the baseline length using a linear actuator, and resulting active and passive forces were measured up to 24% total elongation.

Western blot

Tissue patches were lysed for 30 min on ice in 100 μl buffer consisting of 87% RIPA, 10% glycerol, 5mM EDTA, 3% SDS and 1X Halt Protease and Phosphatase Inhibitor Cocktail (Thermo). During lysis, mechanical disruption was applied every 5 min using a disposable plastic pestle. Tissue lysates were

cleared by centrifugation at 15,000 *g* for 10 min. After determining protein concentration with a standard BCA assay, 30 µg of protein were run in each lane of a 4-15% gradient SDS-PAGE gel (Bio-Rad). Wet transfer (20% methanol) was applied at 100V for 1 hour to immobilize proteins to 0.22 µm nitrocellulose scaffolds (Santa Cruz). Nitrocellulose scaffolds were then carefully cut according to molecular weight and blocked in 5% BSA. Primary antibodies used were Nav1.5 (1:2000, generous gift of Dr. Peter Mohler[5]), Sarcomeric α-actinin (1:5000, Sigma[1]), Kir2.1 (1:1000, Alomone Labs[6]), β-tubulin (1:10,000, Abcam[7]), Connexin-43 (1:5000, Abcam[8]). Secondary antibodies used were horseradish peroxidase-conjugated goat anti-rabbit and anti-mouse (1:5000 and 1:20,000 respectively, Sigma). Bands were visualized using x-ray film or ChemiDoc imaging system (Bio-Rad) and densitometry was performed using ImageJ.

Engineering of cardiac "micro-patch" tissues for conditioned media studies

Ring-shaped frames were punched out of a piece of nylon fabric (Cerex Advanced Fabrics) using two circular (7.9 mm and 5.6 mm diameter) punchers. PDMS (~250 µl) was poured into each well of 24-well plates and allowed to cure (4 hours, 80 °C). PDMS-coated wells were sterilized using 70% ethanol, and a ring-shaped circular nylon frame was pinned in each well (Fig. S3). To form engineered tissues, 35 µl of mESC-CM/gel mixture (Table S3) at a density of $2x10^6$ cell /ml was added to the central portion of each ring and evenly distributed using a pipet tip to allow for the gel solution to infiltrate into the nylon mesh. The gel solution was allowed to polymerize for 45 min at 37 °C, and cultured in 1 ml of serum-free N2B27 medium (Table S4) for up to 14 days. During culture, the cell-gel solution compacted to form a 3D cardiac micro-patch anchored to the circular nylon frame.

Use of conditioned media for micro-patch culture

Cardiac fibroblasts (passaged once) were plated in serum-containing medium (Table S2) at a density of 400 cells/mm^2 on gelatin-coated T-75 tissue culture flasks (3 x 10^6 cells per flask). After 48 hr, plating medium was replaced with serum-free N2B27 medium (10 ml per flask) that was used for conditioning.

Each 48 hr, CF-conditioned media was collected in 15ml conical tubes and replaced with fresh serum-free medium. After removal of any suspended cells and debris by centrifugation (500 *g* for 10 mins), conditioned media was used directly for micro-patch culture or stored at 4 °C for subsequent use. Conditioned media was collected in this manner from the same flasks of cells three times (over 6 days in culture). No decrease in CF density was observed during the conditioning. For selected experiments, proteins in conditioned media were deactivated by trypsin digestion (25 µg/ml, 2 hr, 37 °C) followed by heating (95 °C, 20 min)[9]. For small molecule experiments, micro-patches were pre-conditioned with small molecule inhibitor drugs (Table S5) for 4hrs followed by the addition of the CF-conditioned medium containing same concentration of inhibitor drug (Fig. S3B). The conditioned media with drug was then refreshed every other day until structural and assessment on day 14.

Video analysis of spontaneous beating amplitude in micro-patches

Three, 10, and 14 days after the introduction of conditioned medium, 24-well plates containing micro-patches were mounted on a standard fluorescence microscope with live cell imaging chamber heated to 37°C and perfused with 5% CO_2. For each micro-patch, 5 seconds of video at a frame rate of 25 fps was captured at 4x magnification using a 1.3-megapixel eyepiece camera. Video files were then analyzed by a custom program written in Python. Specifically, video files were loaded into main memory frame-by-frame and converted into 8-bit greyscale images. Due to the relatively low cell seeding density and homogenous nature of the micro-patches, we could not reliably apply classical video-edge detection technique[10] to measure contraction amplitude. Instead, we treated each cell or cluster of cells within micro-patch as a separate region that beats independently of its neighbors, and used greyscale intensity fluctuations as indicators of beating amplitudes in different regions of the micro-patch. Specifically, the time-varying greyscale signal intensity recorded from each pixel was detrended to remove artifactual baseline variation, and amplitude of the fluctuation was computed as a scalar representation of contraction amplitude for that pixel. The average contraction amplitude of each video was determined by averaging the contraction amplitudes over all pixels.

Assessment of MEK-ERK signaling in mESC-CM patches

Pure mESC-CM patches with larger volume (120 uL) and cell density (5 x 10^6 per mL) compared to micro-patches (35 uL, 2 x 10^6 cells per mL) were generated to obtain sufficient cellular protein for Western blot analysis. Patches were cultured for 7 days in unconditioned, adult CF-conditioned, or fetal CF-conditioned serum-free media, or in media supplemented with 15% FBS. Media with or without MEK inhibitor PD0325901 (1 μM) was replenished every other day. Protein isolation and Western blots were performed as previously described for co-cultured patches. Blots were probed for antibodies against ERK1/2 phosphorylated at Thr202/Tyr204 (Cell Signaling Technology #9101, 1:1000 dilution), total ERK1/2 (Cell Signaling Technology #4695, 1:1000 dilution), and GAPDH (Santa Cruz #SC-32233, 1:1000 dilution).

Immunofluorescence

All patch samples were fixed in 2% PFA, permeabilized in 0.1% triton-X, then blocked in a solution of 0.8% BSA + 20% chick serum. Primary antibodies were applied at a concentration of 1:200 - 1:400 in blocking solution at 4 °C overnight. Alexa-Fluor conjugated secondary antibodies and DAPI were applied at a concentration of 1:400 at room temperature for 2 hours.

Analysis of CM spreading in micro-patches

Confocal microscopy (Leica SP5) was used to obtain image stacks from each micro-patch stained with sarcomeric α-actinin and DAPI at 1μm resolution over a total of 12μm depth. Three 20x fields of view were taken per micro-patch. Image stacks were then flattened and analyzed using a custom program written in Matlab. In brief, each color channel was converted to 8-bit greyscale, despeckled, and subjected to thresholding such that only cellular areas (Actn2) or nuclei (DAPI) were shown. Cellular nuclei in each image were counted by using an algorithm based on Hough transformation to recognize partially overlapping or closely-spaced nuclei ("Detect circles with various radii in grayscale image via Hough

Transform" – Tao Peng, MATLAB Central File Exchange). The total cellular area in each channel was then divided by the number of nuclei to measure the amount of cell spreading taking place in each micro-patch.

Monolayer culture of mESC-CMs

Freshly dissociated mESC-CMs were plated onto Aclar® films (Electron Microscopy Sciences) pre-coated with 15 µg/mL fibronectin (Sigma) for 1 hour at 37 °C, as previously described[1]. For analysis of cell spreading in response to CF paracrine factors, 0.1 x 10^6 cells were seeded into each well of a 12-well plate. For Western blot experiments, 0.9 x 10^6 cells were seeded into each well of a 6-well plate. mESC-CMs were maintained in indicated media for 4 days. Immunostaining and Western blot were performed as described for mESC-CM tissues.

Supplementary Figures

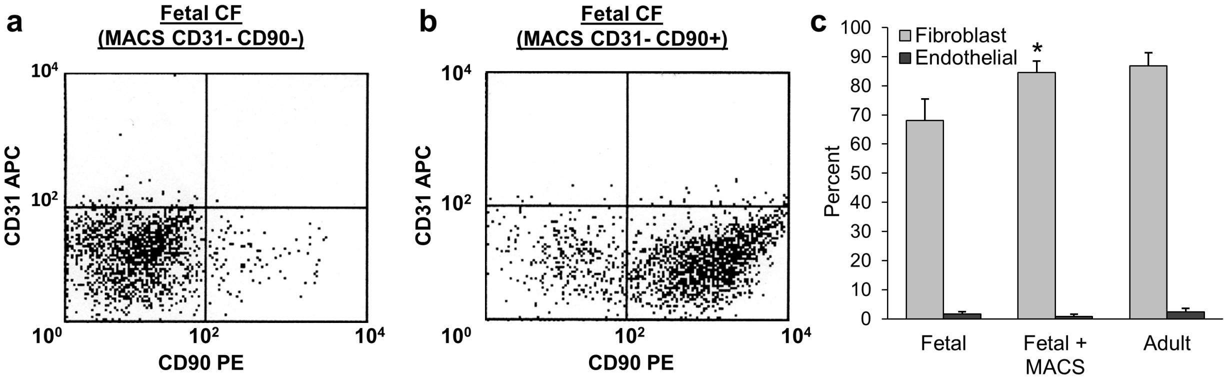

Figure S1: FACS analysis of magnetically sorted fibroblasts.

(**a-b**) Representative FACS scatter plots of fetal cardiac cells after separation by MACS into (a) CD31-/CD90- and (b) CD31-/CD90+ fractions. (**c**) Percent of cardiac fibroblasts (CD31-/CD90+) and endothelial cells (CD31+/CD90+) in fetal and adult cardiac cells before MACS and fetal cells after MACS purification (N = 4 per group). * significantly different from Fetal (non-MACS) group.

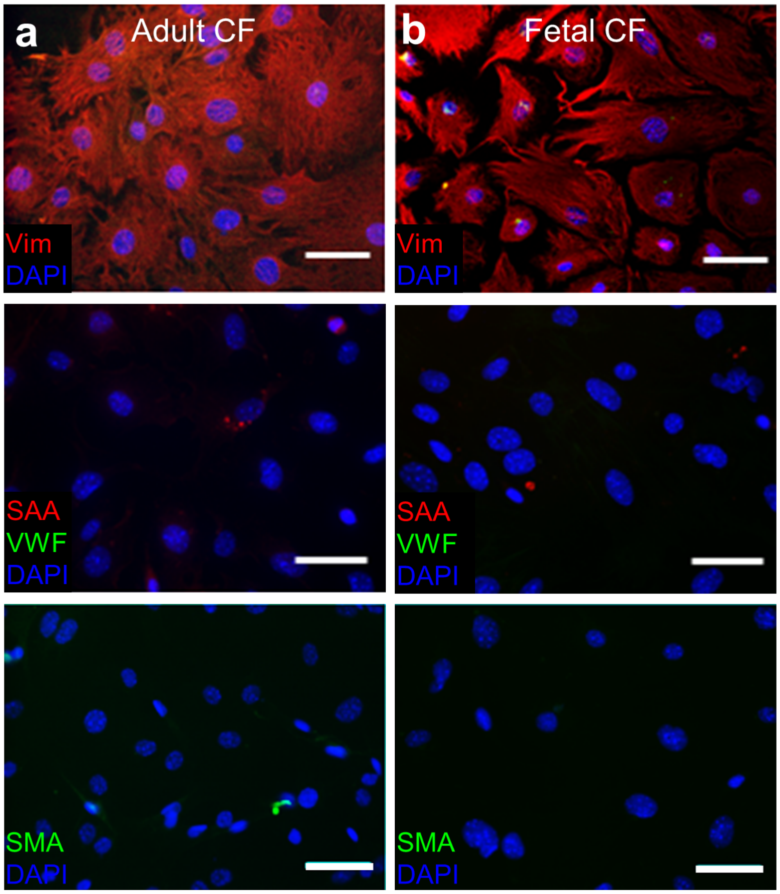

Figure S2. Phenotypic characterization of cultured fetal and adult cardiac fibroblasts.

(**a-b**) Representative isolated cardiac fibroblasts from adult (a) or fetal (b) hearts stained for vimentin (Vim, top row), sarcomeric α-actinin (SAA) and von willebrand factor (VWF, middle row), or smooth muscle actin (SMA, bottom row). All samples were counterstained for nuclei (DAPI); Scale bars: 20 µm.

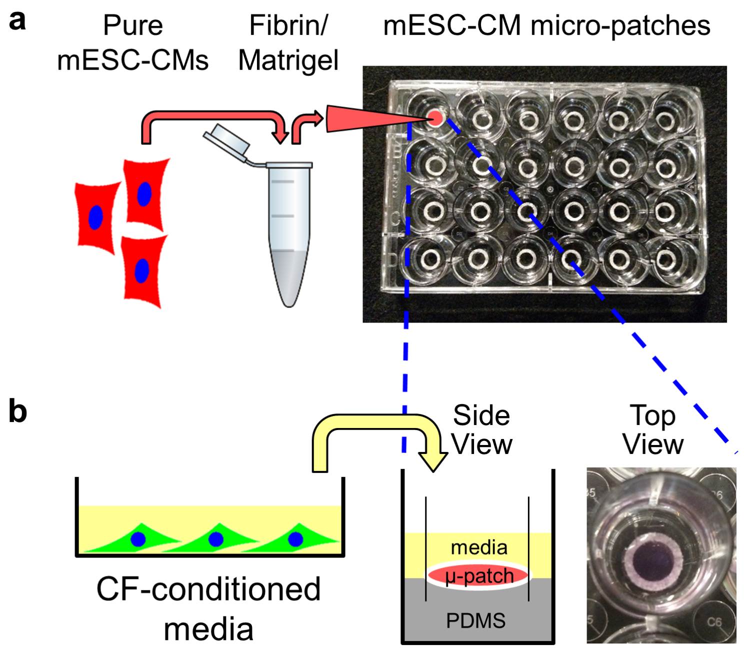

Figure S3. Cardiac "micro-patches" for analysis of CF-conditioned media on mESC-CMs cultured in 3D environment.

(**a**) Purified mESC-CMs are embedded in fibrin-based hydrogel and cast into nylon ring-shaped frames within a 24-well plate. (**a**) mESC-CM micro-patch is held within each well by small pins inserted through the frame into cured PDMS at the bottom of the well. Culture media is conditioned by CFs for 48 hours before being added to micro-patch well and replaced by fresh conditioned media every other day for up to 2 weeks.

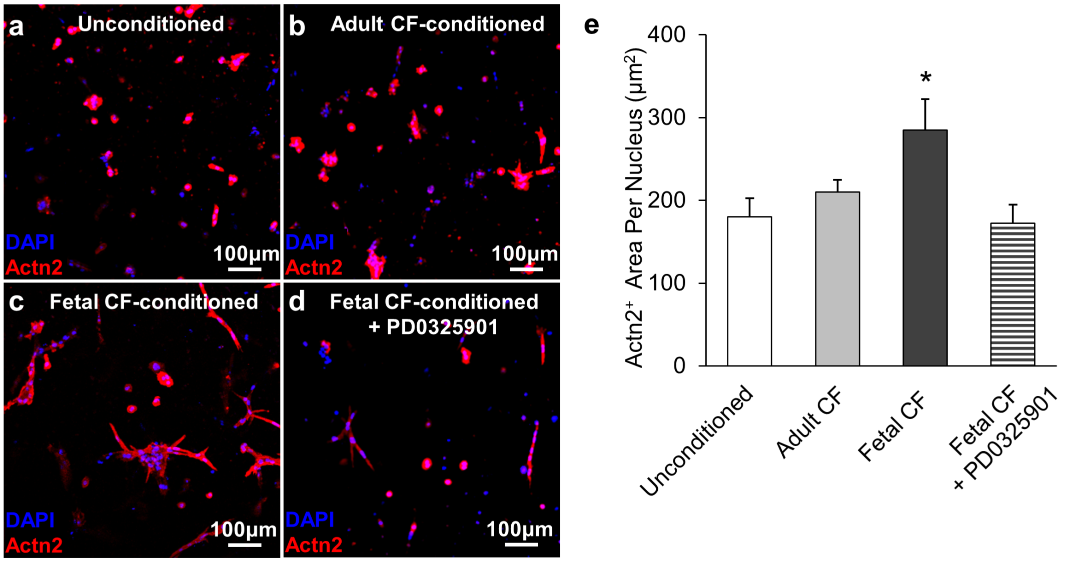

Figure S4. Effects of CF paracrine factors and MEK-ERK inhibition on size of mESC-CMs in micro-patches.

(**a-d**) Representative immunostainings of cardiac micro-patches in control (unconditioned) media (a) or media conditioned for 2 weeks by adult CFs (b) or fetal CFs without (c) or with (d) MEK inhibitor PD0325901, stained for DAPI and sarcomeric α-actinin (Actn2). (**e**) Actn2$^+$ area per nucleus from immunostaining images was quantified as the index of cell size. *, significantly different from all other groups; N = 3 micro-patches per group.

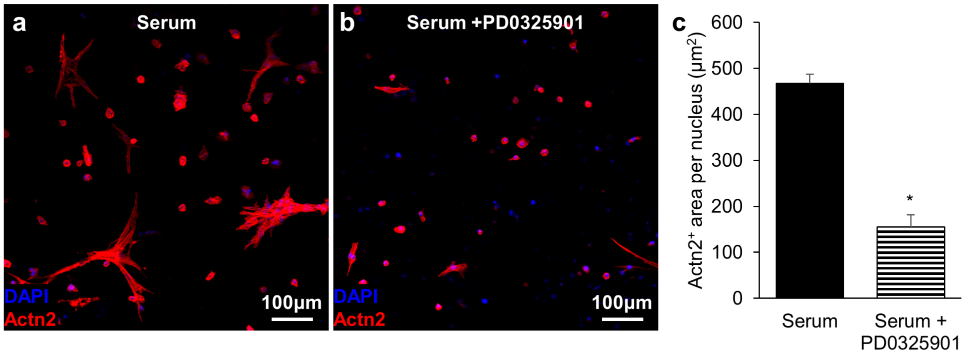

Figure S5. Effects of MEK-ERK inhibition on mESC-CM micro-patches in serum-containing media.

(**a-b**) Representative immunostainings of cardiac micro-patches cultured for 2 weeks in 15% FBS media without (a) or with (b) MEK inhibitor PD0325901, stained for DAPI and sarcomeric α-actinin (Actn2). (**c**) Actn2$^+$ area per nucleus from immunostaining images was quantified as the index of cell size. *, significantly different from micro-patches without PD0325901; N = 6 micro-patches per group.

Supplementary Tables

Reagent	Quantity
Tissue culture water	1 Liter
NaCl	68.0 g
HEPES	47.6 g
NaH_2PO_4	1.38 g
Glucose	6.0 g
KCl	4.0 g
$MgSO_4 \cdot 7H_2O$	2.05 g

Table S1: 10x ADS buffer

Reagent	Quantity	Vendor / Item number
Embryomax ES DMEM	425 ml	Millipore / SLM-220-B
Fetal bovine serum	75 ml	Atlanta Biologicals / E1030
MEM NEAA	5 ml	Stemcell Technologies / 07600
Sodium Pyruvate	5 ml	Stemcell Technologies / 07000
L-glutamine	5 ml	Sigma / G7513
ES-qualified 2-mercaptoethanol	0.5 ml	Millipore / ES-007-E
Gentamicin	0.5 ml	Gibco / 15750-060

Table S2: Serum-containing medium

Reagent	Final Concentration	Vendor / Item number
Fibrinogen solution (10 mg/ml in DPBS)	2 mg/ml	Sigma / F8630
2X DMEM	1X	Gibco / 31600-034
Matrigel	10% (v/v)	BD / 354234
Thrombin	0.8 U/ml	Sigma / T6634

Table S3: Composition of engineered tissue hydrogel

Reagent	Quantity	Vendor / Item number
DMEM/F-12	225 ml	Gibco / 11330-032
Neurobasal medium	225 ml	Gibco / 21103-049
N2 supplement	5 ml	Gibco / 17502-048
B27 supplement	10 ml	Gibco / 12587-010
BSA fraction V	34 ml	Gibco / 15260-037
MEM NEAA	5 ml	Stemcell Technologies / 07600
Sodium Pyruvate	5 ml	Stemcell Technologies / 07000
L-glutamine	5 ml	Sigma / G7513
Gentamicin	0.5 ml	Gibco / 15750-060
6-Aminocaproic acid	0.5 g	Sigma / A2504

Table S4: Serum-free N2B27 medium

**Small Molecule**	**Target Inhibited**	**Concentration**
CT04	Rho	0.25 μg/ml
Y27632	ROCK	10 μM
SP600125	JNK	10 μM
SB203580	p38 MAPK	4.5 μM
AG490	JAK2	10 μM
CP690550	JAK1/2	5 μM
U73122	PLC	5 μM
Chelerythrine Chloride	PKC	5 μM
PD0325901	MEK1/2	1 μM

Table S5: Small molecule inhibitor drugs

Supplementary Video Legends

Supplementary Video 1: Spontaneous contractile activity in pure mESC-CM micro-patches cultured for 14 days in unconditioned serum-free media (top right), or in serum-free media conditioned for 48 hours by adult (bottom left) or fetal (bottom right) cardiac fibroblasts. Scale bar = 100 µm.

Supplementary References

1 Liau, B., Christoforou, N., Leong, K. W. & Bursac, N. Pluripotent stem cell-derived cardiac tissue patch with advanced structure and function. *Biomaterials* **32**, 9180-9187 (2011).

2 Davies, M. P. *et al.* Developmental Changes in Ionic Channel Activity in the Embryonic Murine Heart. *Circulation Research* **78**, 15-25 (1996).

3 Christoforou, N. *et al.* Induced pluripotent stem cell-derived cardiac progenitors differentiate to cardiomyocytes and form biosynthetic tissues. *PloS one* **8**, e65963 (2013).

4 Zhang, D. *et al.* Tissue-engineered cardiac patch for advanced functional maturation of human ESC-derived cardiomyocytes. *Biomaterials* **34**, 5813-5820 (2013).

5 Hund, T. J. *et al.* A βIV-spectrin/CaMKII signaling complex is essential for membrane excitability in mice. *The Journal of Clinical Investigation* **120**, 3508-3519 (2010).

6 Milstein, M. L. *et al.* Dynamic reciprocity of sodium and potassium channel expression in a macromolecular complex controls cardiac excitability and arrhythmia. *Proceedings of the National Academy of Sciences* **109**, E2134–E2143 (2012).

7 Bian, W. & Bursac, N. Soluble miniagrin enhances contractile function of engineered skeletal muscle. *The FASEB Journal* **26**, 955-965 (2012).

8 Mahmoodzadeh, S. *et al.* 17β-Estradiol-induced interaction of ERα with NPPA regulates gene expression in cardiomyocytes. *Cardiovascular Research* **96**, 411-421 (2012).

9 Pedrotty, D. M., Klinger, R. Y., Kirkton, R. D. & Bursac, N. Cardiac fibroblast paracrine factors alter impulse conduction and ion channel expression of neonatal rat cardiomyocytes. *Cardiovascular Research* **83**, 688-697 (2009).

10 Shimizu, T. *et al.* Fabrication of Pulsatile Cardiac Tissue Grafts Using a Novel 3-Dimensional Cell Sheet Manipulation Technique and Temperature-Responsive Cell Culture Surfaces. *Circulation research* **90**, e40-e48 (2002).
